# Voluntary wheel running: patterns and physiological effects in mice

**DOI:** 10.1590/1414-431X20187830

**Published:** 2018-12-10

**Authors:** G. Manzanares, G. Brito-da-Silva, P.G. Gandra

**Affiliations:** Departamento de Bioquímica e Biologia Tecidual, Instituto de Biologia, Universidade Estadual de Campinas (UNICAMP), Campinas, SP, Brasil

**Keywords:** Voluntary wheel running, Mouse, Exercise, Skeletal muscle, Endurance adaptation

## Abstract

Exercise can prevent and improve the pathophysiology of diseases and promote healthy aging. Thus, understanding the mechanisms that regulate the beneficial effects of exercise may lead to the development of new strategies to enhance quality of life and to counteract chronic diseases. Voluntary wheel running is an interesting model to study the effects of exercise in mice. Compared to forced treadmill exercise, voluntary wheel running presents several advantages such as: 1) running pattern is similar to natural running behavior of mice; 2) it is performed under non-stressed conditions, according to the rhythmicity of the animal; 3) it does not require direct interference from the researcher, and can be easily applied in long-term studies. Mice run spontaneously when given access to running wheels, for a total distance of ∼4 to 20 km per day and a total activity time of ∼3 to 7 hours a day. Hence, voluntary wheel running can result in robust endurance-like adaptation in skeletal and cardiac muscles and protect from sarcopenia. However, due to the lack of control over exercise parameters in voluntary exercise models, it is important for the researcher to understand the patterns and variability of wheel running in mice, as well as the factors that can affect voluntary running activity. Overall, voluntary wheel running in mice is a very interesting approach to study the chronic adaptation to exercise, analyze the effects of exercise, and test exercise capacity in different experimental models.

## Introduction

Physical exercise can prevent and ameliorate the pathophysiology of different diseases and increase health span (1). However, exercise tolerance can be decreased in chronic diseases and aging. Therefore, understanding the mechanisms regulating the gain or loss of muscle function and exercise capacity may lead to the development of new strategies to enhance the beneficial effects of exercise and to counteract chronic diseases. Voluntary wheel running in mice is a widely used model to study exercise and behavior (2). Laboratory mice run spontaneously when they have access to running wheels. Remarkably, this behavior is also observed in feral wild mice when running wheels are placed in nature (3). Voluntary wheel running seems to satisfy playing, escaping, or metabolic related drives, consisting of a rewarding behavior and not a stereotypic behavior that can result from environmental restriction and devoid of any goal or function (3–5). Voluntary wheel running can be used to evaluate changes in behavior and exercise capacity, such as what occurs in disease models (6) or in genetically modified mice (7,8). Mice spontaneously have relatively high activity levels per day on wheels (i.e., ∼3 to 7 h and ∼4 to 20 km per day) (9–11). Therefore, voluntary wheel running can also be used to study the adaptive responses to exercise (10) and to determine the effects of exercise on diseases (6,12) or aging (13).

Mice running behavior in voluntary wheels is more similar to the natural running pattern than forced treadmill exercise (9). Also, voluntary wheel running is performed under non-stress conditions, as it does not require a negative stimulus (such as electric shock or touching the animals, which are used in forced treadmill running), and does not interfere in the normal nocturnal-diurnal rhythmicity of the animal (8). Another advantage of voluntary wheel running is that, since no direct intervention from the experimenter is required, it can be easily used in long-term studies (i.e., 12 months or lifelong exercise). The main drawback of voluntary wheel running is that the researcher does not have control of the total exercise amount and intensity performed. One way to overcome this limitation is to understand the patterns and variability in voluntary wheel running activity in mice, as well as the factors affecting voluntary running activity.

In this mini-review, we describe voluntary wheel running patterns in mice and its effects on exercise adaptation. Voluntary wheel running activity is strongly influenced by sex, strain, age, the design of the running wheel, diet, and environment conditions, which consequently may impact the voluntary running period. To isolate each individual variable in mouse voluntary running, we limited some of the discussion to studies with C5BL/6J mice. The C57BL/6J mouse is the most widely used inbred strain, and it has been shown to be highly motivated to run at relatively high speeds when running wheels are provided (9,14).

## Exercise behavior in the running wheel

### Voluntary wheel running pattern

Mouse voluntary wheel running activity is almost entirely accomplished during the dark phase of the 12/12 h light-dark cycle (9,15–17). Interestingly, mice start to run immediately after the beginning of the dark cycle (9,15). Running activity peaks during the initial 2 to 3 h of the dark-cycle, and decreases progressively (9,15). Thus, the total running time and distance are reduced during the second half of the dark-cycle, while the running speed seems to remain unchanged (9,15). The activity pattern on the running wheel is composed of several running bouts of ∼150 s each, separated by short breaks of ∼30 s (9,15). The running speed and total running distance per day varies for each mouse strain. For the C57BL/6J, the reported average running speed ranges from ∼1.5 to 3.0 km/h (9,10,18–20), while maximum running speed ranges from ∼2.7 to 4 km/h (9,10). In general, studies report that C57BL/6J mice run voluntarily for a total activity time of ∼3 to 7 h per day and a total distance that can range from ∼4 to 20 km per day (10,15,18,19,21). The differences in these reported running speeds and total running time and distance per day are likely due to factors that are known to affect voluntary running pattern such as age, sex, running wheel design, and environmental variables.

Voluntary wheel running in mice is characterized as being intermittent, which can resemble interval training in humans. However, the total amount of time and distance run per day is relatively high, indicating that mice perform a significant amount of activity on running wheels.

### Types of running wheels

**Figure 1:**
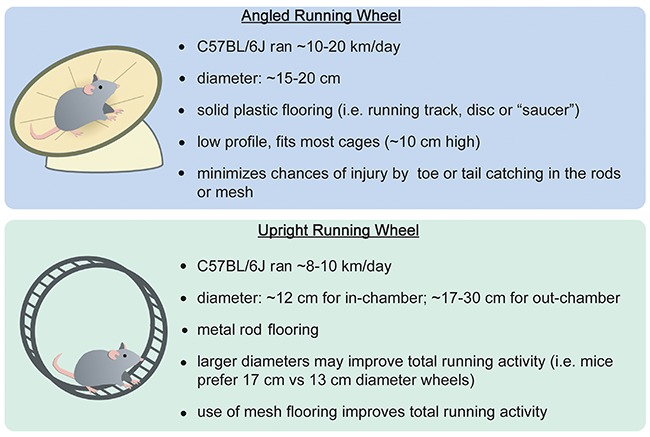
Characteristics of the different types of running wheels for mice. The design of the running wheel can affect total voluntary running activity in mice. Mice present higher voluntary running activity in angled wheels compared to upright wheels.

A common method to record total distance, time, and speed on running wheels is to attach a magnetic sensor that quantifies the number of revolutions and the total time of running activity per day (a bicycle computer is typically used for this purpose) (9,11). A more sophisticated apparatus connected to an analysis software can be used when detailed activity parameters are needed, such as number and duration of each running bout and the dark-light cycle activity patterns. Currently, there are several mouse wheel activity tracking systems commercially available. There are two major types of running wheels used in mouse studies, upright wheels and angled wheels (also refereed as running tracks or discs; Figure 1). Most studies use in-chamber upright wheels with a diameter of ∼12 to 13 cm (10,15,17,22,23). However, mice prefer larger upright wheels, favoring wheels with 17.5 cm diameter over 13 cm setups, suggesting that wheels with larger diameters may improve total running activity (4). Mice were also shown to prefer a mesh flooring to metal rods in upright wheels (4). It is important to consider that larger upright metal wheels, ∼17 to 35 cm in diameter, may present larger resistance than smaller wheels, and may affect mice running pattern (larger wheels are usually separated from the cage) (24). A resistance inferior to 1 g was suggested as a reference to be used with mice for a very low resistance wheel or free-spinning wheel (25).

In the past decade, activity-tracking systems using angled wheels became commercially available for mouse research, and their use has become more frequent. One of the advantages of the angled wheel is its small height (∼10 cm) that allows its use in regular mouse cages. The diameter of the angled wheels varies from ∼12 to ∼40 cm with a track width of ∼3.5 cm (9,26) (e.g. Fast-Trac Wheel, Model BSK3251, diameter: 12.4 cm, track width: 3.6 cm; Lafayette Instrument, USA). Also, the angled wheel has a solid plastic surface that mice prefer over the rods of upright wheels (27) and that can minimize the chances of injury due to toe or tail catching. Accordingly, a study showed that mice run more in angled wheels, when both wheel types were available in the cage (27). It has also been proposed, that in angled wheels, mice run on a planar locomotion pattern that better resembles their natural running gate pattern (26). In upright wheels, mice do not run in a planar pattern, which can potentially cause ventral arching of the spine and hyperflexion of the tail, altering the animal locomotor abilities (16). Yet, we are unaware of studies describing the incidence of these postural alterations or the specific conditions that induce them. As for the angled running wheels, one possible disadvantage is that the mouse is constantly running around a bend and on a slight incline, and it is not clear if this can affect their running stance and overall running pattern.

The design of the running wheel can have great effects on mouse running activity and patterns. Voluntary running activity is increased on plastic angled wheels when compared to upright metal wheels. When using upright wheels, mice prefer larger diameters and mesh floorings. However, larger and heavier wheels can result in increased resistance, which may also influence mice running patterns.

### Total voluntary running distance per day in different running wheels

**Figure 2:**
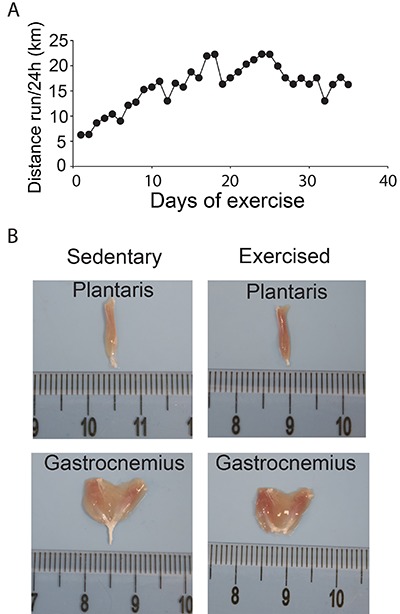
Total distance run per day, starting at ∼10 weeks of age (*A*). Plantaris and gastrocnemius muscles from a sedentary mouse (left) and an exercised mouse, right (*B*). Note the darker reddish color of the muscles from the exercised mouse. The exercised mouse ran a total distance of ∼500 km in 5 weeks. Data and photographs are unpublished observations from Manzanares G, Brito-da-Silva G, and Gandra PG. Data were obtained in accordance with the Brazilian Society of Laboratory Animal Science guidelines and approved by the Institutional Committee for Ethics in Animal Use (CEUA/IB/UNICAMP, #4762-1/2017).

In studies with adult C57BL/6J mice, the mice tend to cover longer distances per day in angled running wheels compared to upright wheels. Two studies in which male C57BL/6J mice had free access to angled disc wheels reported a total running distance of ∼20 km per day (11,28). In another study with angled wheels, male C57BL/6J mice ran an average peak distance of ∼10 km per day and females ran ∼16 km per day (9). In our laboratory, we have been observing that in angled wheels, male C57BL/6J mice can run an average of ∼17 km per day during the third week of voluntary exercise and an average of ∼12 km per day during the first three weeks of exercise (Figure 2). Conversely, in upright running wheels, studies with adult male C57BL/6J mice have reported peak running distances of ∼8.5 to 10.0 km per day during the third week of voluntary wheel running (10,15) or an average distance of ∼4 to 8 km per day during the first three weeks of exercise (10,18,21). Running parameters of different studies using upright running wheels and angled wheels are presented in Table S1.

It is clear that the design of mouse running wheels affects voluntary running parameters. So far, the results indicate that the speed, total running distance, and time run per day are significantly higher when angled wheels are used.

### Changes in running pattern throughout a period of voluntary wheel running

The use of running wheels does not require a familiarization period; still, some studies adopt 1 to 2 days of familiarization with a locked wheel before starting the exercise protocol. During the first weeks of exposure to running wheels, the total running distance per day increases progressively, reaching a plateau after 2 to 5 weeks (9,15,29,30). Similarly, the total time spent running also increases, peaking after ∼5 to 16 days (9). After reaching a peak, the total running distance and time per day can decline slightly during the following weeks (9,10,29). The total distance run per day can have an average day-to-day variability ranging from ∼17 and 25% (Figure 2). The average running speed increases during the first 3 weeks. After this period, running speed reaches a plateau that is maintained, with no apparent day-to-day variations, until it starts to decrease as the animals age (9,15). Regarding individual running bouts, their duration increases during the first 10 to 15 days of exercise reaching averages of approximately ∼150 to 200 s (9).

Overall, running activity increases during the first 3 weeks of voluntary wheel running. Hence, it is important to consider the amount of time mice were exposed to wheels when comparing running parameters between different studies. Changes in behavior, as well as physiological adaptations, are likely responsible for the increased voluntary running activity during the initial weeks of exposure to the wheel.

### Differences in voluntary wheel running pattern between female and male mice

It has been consistently observed that female mice run more when given access to wheels than their male counterparts. This higher voluntary running performance in females is observed for young adult and aged mice of different strains (9,13,15,21,31,32). Female C57BL/6J mice run average distances ∼40 to 50% longer than male mice (10 to 16 weeks old) (9,21). Similar differences in running activity between females and males were observed for FVB/NJ mice, suggesting that sex differences in running behavior are preserved in different genetic backgrounds (21). In one study, the difference in running activity between sexes resulted from female mice running for longer periods during the second half of the night (9). Regarding running speed, it was reported that females ran at higher average speeds only during the first 3 weeks of a voluntary running protocol (9,21).

Female mice present a higher day-to-day variability in total voluntary running activity compared to males (9,15). The mean day-to-day variation in the total time spent running in a running period of 35 days was ∼25% for females compared to ∼17% for males (9). It is likely that the day-to-day variability in female mice is affected by the estrous cycle. Female mice were observed to run more immediately after the pro-estrus phase (7.8 km per day) and less after the diestrus (5.5 km per day) (29).

Sex influences voluntary running and female mice run longer distances than males in running wheels. This suggests that the use of females could be advantageous if one is interested in imposing greater exercise stimuli on the animals. Yet, it is not completely clear how sex affects the adaptive responses to a period of voluntary running. Additionally, the day-to-day variability in voluntary running activity in males and females must be considered, especially when studying short-term exercise interventions.

### Voluntary wheel running protocols

Voluntary wheel running can be used to test exercise capacity or to induce adaptive responses to exercise (Supplementary Table S1). The use of voluntary running to study the acute effects of exercise is limited because it is impossible to control the speed and duration of the activity. In order to test changes in exercise capacity, voluntary wheel running can be followed for 5 to 7 days, which minimizes the impact of the day-to-day variations in running patterns and avoids the confounding effects of physiological responses to longer exercising periods (8). For example, running wheels can be used to quantify exercise capacity in genetically modified mice or different disease models (6–8).

To induce physiological adaptations to exercise, a minimum amount of stimulus is necessary. Voluntary wheel running in mice is characterized by a progressive increase in running distance, reaching relatively high activity levels after ∼3 weeks (from <5 km a day to ∼10 to 20 km a day (9,11); Figure 2). Voluntary running protocols lasting from 3 to 5 weeks have resulted in fairly robust adaptations in skeletal muscle and heart (10,19) (the adaptive responses to wheel running are discussed in the next sections; see Figure 2 for examples of qualitative changes in skeletal muscle with voluntary running). Also, long-term (lasting several months) or life-long voluntary wheel running has been used to study the effects of exercise in aging (23,33).

Another possibility is the use of loaded running wheels, as a form of resistance exercise. In this case, wheels are equipped with a servo brake (commercially available or alternatively using a home-made modified wheel system) (25,34,35). Loaded voluntary running protocols usually use a progressive increase in resistance loads throughout the weeks. Interestingly, the addition of resistance loads on the wheel do not seem to affect the total distance run per day and the running speed compared to mice running without resistance. Total time run per day and speed decreased only when resistance is higher than 9 g (34).

## Adaptation to voluntary wheel running

### Skeletal muscle adaptation to voluntary wheel running

Mice subjected to a period of voluntary wheel running can present better endurance capacity as indicated by a significant increase (∼90%) in the time to exhaustion and VO_2max_ on treadmill tests (19,36). Accordingly, after ∼3–4 weeks of voluntary wheel running, skeletal muscles (gastrocnemius, tibialis anterior, triceps) of male C57BL/6 mice, show enhanced citrate synthase activity (19,22), marked increase in the abundance (∼2 to 6-fold), and activity of oxidative phosphorylation complexes (19), higher levels of proteins related to mitochondrial biogenesis (i.e., PGC-1α, TFAM), and related lipid and carbohydrate oxidation (19). Upregulation of PGC-1α and mitochondrial proteins expression, as well as increased activity of metabolic enzymes were also reported in the skeletal muscle after 4 to 8 weeks of voluntary wheel running (32,37). Another endurance-type adaptation observed after 4 weeks of voluntary running was an increased capillary density and capillary-to-fiber ratio in the plantaris muscle of male C57BL/6J mice (38). Voluntary running also causes changes in the fiber type composition of skeletal muscle (10,30,38). In the soleus, gastrocnemius, and plantaris muscles, voluntary running induces increases in the relative composition of myosin heavy chain (MHC) type IIa and decreases in MHC-IIx and -IIb (10,30,38). The diaphragm also appears to adapt to voluntary running, as indicated by enhanced levels of diaphragm mass and MHC-IIa composition (30). Hind limb skeletal muscle mass does not significantly change after voluntary running in young healthy mice (10,19,30). In old mice, skeletal muscle mass has been reported to be augmented after long-term or loaded wheel running (25,34).

### Heart adaptation to voluntary wheel running

Mice subjected to voluntary running in wheels consistently have a bigger heart mass compared to sedentary mice (9,10,19,21,27,34). In male C57BL/6J mice exercised in running wheels, cardiac mass was reported to increase by ∼5–10% after 20 to 21 days (19,21), and by ∼15% after 4 weeks of wheel running (10). Resting heart rate also decreased after 20 days of wheel running, confirming that 20 days of wheel running results in adaptations in the heart (39). Notably, studies analyzing the effects of sex on cardiac hypertrophy induced by voluntary running observed that the increase in heart mass is more pronounced in exercised female mice (9,21). For example, when female and male C57BL/6J mice were compared for changes in cardiac mass, it was observed that the cardiac mass of exercised females was ∼16% higher than in sedentary mice while in males this increase was ∼5% (9,21). Even when the heart mass was normalized to total amount of wheel activity, which is higher for female mice, a greater increase in heart mass was still observed in females (9,21).

### Total body mass and body composition responses to voluntary wheel running

Testing the effects of voluntary running in body composition has generated mixed results. Some studies did not observe alterations of total body mass of young mice submitted to voluntary wheel running (10,15,21,37,40). Two studies showed a ∼5–10% lower body mass in exercised male C57BL/6J mice compared to sedentary controls (these effects were not observed in female mice) (7,9). In another study, the epididymal fat mass in male C57BL/6 mice was significantly reduced in exercised mice (20 days of voluntary wheel running) (19). However, in a study with female C57BL/6 mice, 6 weeks of voluntary running did not alter the perigonadal fat mass (41). Changes in total body mass, lean mass, and fat mass, as a result of voluntary wheel running, seem to be more pronounced with long-term protocols and in old mice as will be discussed further in this review (13,33).

To sum up, voluntary wheel running results in increased endurance capacity in both female and male mice. In skeletal muscle, adaptations related to mitochondrial biogenesis and increased oxidative capacity can be observed as soon as 20 days of voluntary running. Changes in cardiac mass can also occur after 20 days of voluntary running, and cardiac hypertrophy adaptation to exercise is greater in female C57BL/6J mice.

## Effects of aging on voluntary running exercise

### Aging and voluntary running pattern

Voluntary running capacity declines gradually as mice age. When mice are ∼10 months old, the total wheel running distance can be decreased by ∼45% compared to young mice (5–14 weeks old) (15). When mice are 22–24 months old (roughly corresponding to 70 years in humans), distance run per day is reduced by ∼50% compared to ∼10-month-old mice or by ∼75% compared to young animals (∼2–4 months old) (11,15). Aging also affects the voluntary wheel running patterns. At old age, mice demonstrate a decrease in running speed, and tend to reach their maximal running distance later during the dark cycle (15). Interestingly, during aging, female mice still outperform male mice in voluntary wheel running (13,15,21). Reports show that running distances per day is around 1.5–2-fold higher in 12–15-month-old females compared to male mice (13,21).

### Aging and long-term voluntary wheel running

Long-term voluntary wheel exercise in mice can be used as an intervention to mimic an active lifestyle in humans, allowing the study of the effects of exercise in aging. Voluntary running exercise has been shown to increase the average or median lifespan of mice and rats by ∼10 to 17% while maximum lifespan is not affected (23,42). A study in which 1-year-old mice had access to wheels for one year showed that long-term voluntary exercise protected the animals from age-related gains in total body mass and fat mass, and prevented the loss of lean mass (13). Life-long wheel running was shown to attenuate age-related changes in gene expression in the heart of old mice (i.e., inflammatory and stress response genes; ∼2.75-year-old animals) (23). Also, long-term voluntary resistance running (from 15 to 23 months of age) prevented sarcopenia in hind limb muscles, resulted in hypertrophy in the soleus, augmented heart mass, and increased citrate synthase activity in skeletal muscle of male and female C57BL/6J mice (25).

In sum, although exercise capacity is significantly reduced in old mice, long-term voluntary wheel running still results in exercise adaptations. Voluntary exercise can prevent age-related changes in body composition, avert sarcopenia, and enhance the average life span in mice.

### Genetic background and voluntary wheel running activity

Voluntary wheel running activity in mice is significantly affected by genetic background (14,18). In a study analyzing different inbred mouse strains, it was observed that C57BL/6J mice run significantly more and at a higher average speed, compared to six other strains (14). In contrast, another study showed that C57BL/6J mice had lower voluntary running performance compared to other inbred strains, while C57L/J mice had the highest activity on running wheels (3.77 km and 7.95 km per day, for C57BL/6J and C57L/J mice, respectively) (18). While it appears that voluntary running performance is dependent on genetic background, these studies showed conflicting results for some of the strains tested. These inconsistencies may be due to variations in environmental factors (e.g., housing, wheel type) or differences in mouse age.

### Voluntary wheel running, diet, and circadian rhythm

Voluntary wheel running enhances mouse food intake (by ∼20%) and water consumption (15,17,30,41,43,44). Reciprocally, diet can also affect voluntary wheel running patterns in mice. There are divergent results regarding the effects of a high-fat diet on voluntary running activity. Female C57BL/6J mice under a high-fat diet did not exhibit changes in wheel running activity compared to animals under a regular chow diet or a low-fat diet (29,41). In contrast, 6 weeks of a high-fat diet decreased voluntary running activity in C57BL/6J and DBA/2 male mice (45). In outbred mice selected for high running capacity, a western diet caused an increase in running activity (this was not observed in non-selected lines) (46).

Caloric restriction and time restriction diet interventions have been extensively studied in mice due to their promising effects on the regulation of metabolism and aging. When combined with voluntary wheel running, caloric restriction did not alter total running distance in healthy C57BL/6J mice (although it severely improved voluntary running in *db/db* mice, a model of diabetic dyslipidemia) (47). Nevertheless, caloric restriction was shown to affect the temporal pattern of voluntary running. As previously discussed, voluntary wheel running is performed almost exclusively during the dark-cycle. In a study with rigorously controlled conditions, caloric restriction increased the amount of voluntary wheel running activity occurring during the light-cycle (17). In this same study, when mice were fed on alternate days, voluntary wheel running activity duration was higher during the fasting days, while time-restricted diets did not alter mouse running patterns (17).

Voluntary wheel running can affect the circadian rhythm, which likewise affects the feeding behavior. Access to a running wheel was shown to advance the circadian phases of food intake, as well as increase body temperature and corticosterone secretion (43). Wheel running also phase-advanced the expression of circadian clock genes and clock-controlled genes in the liver and white adipose tissue (43). In skeletal muscle, voluntary wheel running also enhanced the expression levels of certain clock genes (43).

In conclusion, high-fat diet, caloric restriction, and time-restricted diet interventions can alter voluntary running patterns. Additionally, voluntary running can advance the circadian phases, which may affect the organism at different levels, from gene expression to behavior.

## Concluding remarks

In this mini-review, we discussed some of the variables that affect voluntary wheel running activity in mice and the physiological responses to voluntary running, with emphasis on endurance exercise adaptation. Voluntary wheel running is rewarding for mice, and is performed under no psychological stress (3,4), in a running pattern that resembles the natural running behavior for mice (9,26). Differently, the running pattern in forced treadmill running differs from mouse natural running behavior, and is performed under a stressful condition with the use of negative stimuli. The outcomes of voluntary and forced exercise can be markedly different. For example, the gut microbiota is altered differently by voluntary or forced exercise (48): while voluntary exercise improved a mouse model of colitis, forced exercise caused increased inflammation and mortality (49).

Because it is not possible to control exercise stimuli in voluntary wheel running, it is important to consider the different factors that can affect running activity and consequently influence the outcomes of a voluntary running period. Factors such as wheel design, sex, strain, diet, and age can affect voluntary wheel running patterns. Still, mice run voluntarily for a large amount of time per day, which has been shown to induce robust endurance adaptation in skeletal and cardiac muscles after a couple of weeks with access to running wheels. Voluntary wheel running minimizes the stress inflicted on the animals and can be easily implemented. This model can be particularly useful for long-term investigations. Overall, the use of voluntary running wheels is an effective and interesting method to study the effects of exercise in a variety of mouse models.

## Supplementary Material

Click here to view [pdf].
